# Phosphoglycerate kinase 1 as a potential prognostic biomarker in papillary thyroid carcinoma

**DOI:** 10.3389/fphar.2025.1542159

**Published:** 2025-07-23

**Authors:** Xiao-Qing Shi, Xiao-Tong Liu, Xiao-Hui Liu, Hui-Wen Geng, Zhuo Zhang, Wei-Yi Wang, Xing Chen, Xian Zhao, Bo-Lin Jue, Bo Qin, Pei-Pei Liu

**Affiliations:** ^1^ Department of Clinical Laboratory, The First Affiliated Hospital of Zhengzhou University, Key Laboratory of Laboratory Medicine of Henan Province, Zhengzhou, Henan, China; ^2^ Department of Clinical Laboratory, The First Hospital of Yongnian District, Yongnian, Hebei, China; ^3^ Clinical Systems Biology Laboratories, The First Affiliated Hospital of Zhengzhou University, Zhengzhou, Henan, China; ^4^ Department of Neurology, The First Affiliated Hospital of Zhengzhou University, Zhengzhou, Henan, China; ^5^ Department of Neurology, The First Hospital of Lankao, Kaifeng, Henan, China; ^6^ College of Public Health, Zhengzhou University, Zhengzhou, Henan, China; ^7^ Translational Medical Center, The First Affiliated Hospital of Zhengzhou University, Zhengzhou, Henan, China; ^8^ College of Basic Medical Sciences, Xinxiang Medical University, Xinxiang, Henan, China

**Keywords:** papillary thyroid carcinoma (PTC), phosphoglycerate kinase 1 (PGK1), clinicopathological characteristics, recurrence, prognosis

## Abstract

**Backgroud:**

Papillary thyroid carcinoma (PTC) represents a malignant epithelial tumor characterized with a preference for younger individuals. Despite its generally favorable prognosis, PTC still poses considerable challenges, particularly in regards to the propensity for distant metastasis. As a key enzyme in the glycolytic pathway, phosphoglycerate kinase 1 (PGK1) has been linked to the progression of various cancer types. However, its role in PTC remains to be elucidated. This study aimed to investigate the association between PGK1 expression in thyroid cancer tissues and clinicopathological features, postoperative recurrence, and prognosis to provide clinical assessment and intervention reference.

**Methods:**

We investigated the correlation between PGK1 expression and the clinicopathological characteristics, recurrence, and prognosis in 97 PTC patients who underwent surgical treatments between 1 January 2020, and 31 December 2020 in Zhengzhou University First Affiliated Hospital. Besides, we also analysed the correlation of PGK1 expression with the 10-year survival rate of patients with thyroid carcinoma (THCA) in UALCAN database.

**Results:**

PGK1 expression was higher in cancerous tissues than that in adjacent non-cancerous tissues. Further analysis of PGK1 expression across clinicopathological characteristics revealed that patients with poorly differentiated tumors, TNM stages III–IV, lymph node metastasis, and tumor diameter ≥1.0 cm exhibited higher PGK1 expression levels in cancerous tissues. A subsequent 3-year postoperative follow-up was conducted to evaluate the correlation between PGK1 expression and recurrence. During this period, 31.96% of patients experienced recurrence, with higher PGK1 expression correlating with increased recurrence rates. Moreover, patients with higher PGK1 expression in cancerous tissue exhibited a significantly lower survival rate of 79.20% compared to the PGK1-low/medium group. Lastly, age, lymph node metastasis, differentiation degree, TNM stage, and tumor diameter were identified as risk factors for poor prognosis in patients with PTC analyzed by Cox regression.

**Conclusion:**

Our study demonstrated that PGK1 expression may serve as a potential prognostic biomarker of PTC.

## Highlights

• Phosphoglycerate kinase 1 (PGK1) expression was higher in cancerous tissues than in adjacent non-cancerous tissues of papillary thyroid carcinoma (PTC);
• PTC patients with poorly differentiated tumors, TNM stages III–IV, lymph node metastasis, and tumor diameter ≥1.0 cm exhibited higher PGK1 positive expression rates in cancerous tissues;
• PTC patients with higher PGK1 expression are correlated with higher recurrence rates and lower survival rate after 3-year postoperative follow-up;
• Age, lymph node metastasis, differentiation degree, TNM stage, and tumor diameter are risk factors for poor prognosis in patients with PTC.

## 1 Introduction

Papillary thyroid carcinoma (PTC) is a malignant neoplasm that develops from the epithelial cells that line the thyroid follicles (Lam, 2022a; Abe and Lam, 2022). It is characterized by its histological features, which include the presence of papillae and nuclear grooves and the potential for psammoma body formation (Boucai et al., 2024; Nguyen et al., 2015). Recently, the incidence of PTC has increased, with a preference for younger individuals and a higher prevalence in females (Lam, 2022b). Therapeutic interventions for PTC are multifaceted and aim to eradicate the primary tumor, manage potential metastases, and reduce the risk of recurrence, with treatments typically including surgical resection, radioactive iodine therapy, and suppressive thyroid hormone therapy (Filetti et al., 2019; Coca-Pelaz et al., 2020). PTC has challenges despite having a relatively favorable prognosis compared to other malignancies. The potential for local invasion into surrounding tissues and distant metastasis to sites including the lungs and bones is a concern that must be carefully monitored and managed (Yamada et al., 2015; Fonseca et al., 2024). Therefore, accurate diagnosis, staging, and individualized treatment strategies are essential for improving patient survival and quality of life (Xu et al., 2023).

Phosphoglycerate kinase 1 (PGK1) is an essential glycolytic enzyme that catalyzes the conversion of 3-phosphoglycerate (3-PGA) to 1,3-bisphosphoglycerate, which is an essential regulator of cellular energy metabolism (He et al., 2019; Zhang et al., 2023). Previous studies reported that PGK1 expression is consistently associated with the aggressive characteristics of tumor cells, including their proliferation, migration, and invasive abilities (Liang et al., 2020; Ye et al., 2020). Simultaneously, high levels of PGK1 have been observed in numerous malignancies and are associated with a poor prognosis, indicating its potential as a prognostic biomarker and a therapeutic target (Chu et al., 2021; Zhang et al., 2018). Furthermore, the role of PGK1 in cancer cell metabolism is significantly compelling. It is hypothesized to contribute to the Warburg effect, which is the inclination of cancer cells for glycolysis over oxidative phosphorylation, even in the presence of oxygen (Wang et al., 2023; Chen et al., 2023). However, the association between PGK1 and clinicopathological features and the prognosis of patients with PTC is unclear.

This study aimed to investigate the association between PGK1 expression in thyroid cancer tissues and clinicopathological features, postoperative recurrence, and prognosis to provide clinical assessment and intervention reference.

## 2 Materials and methods

### 2.1 General information of patients

**FIGURE 1:**
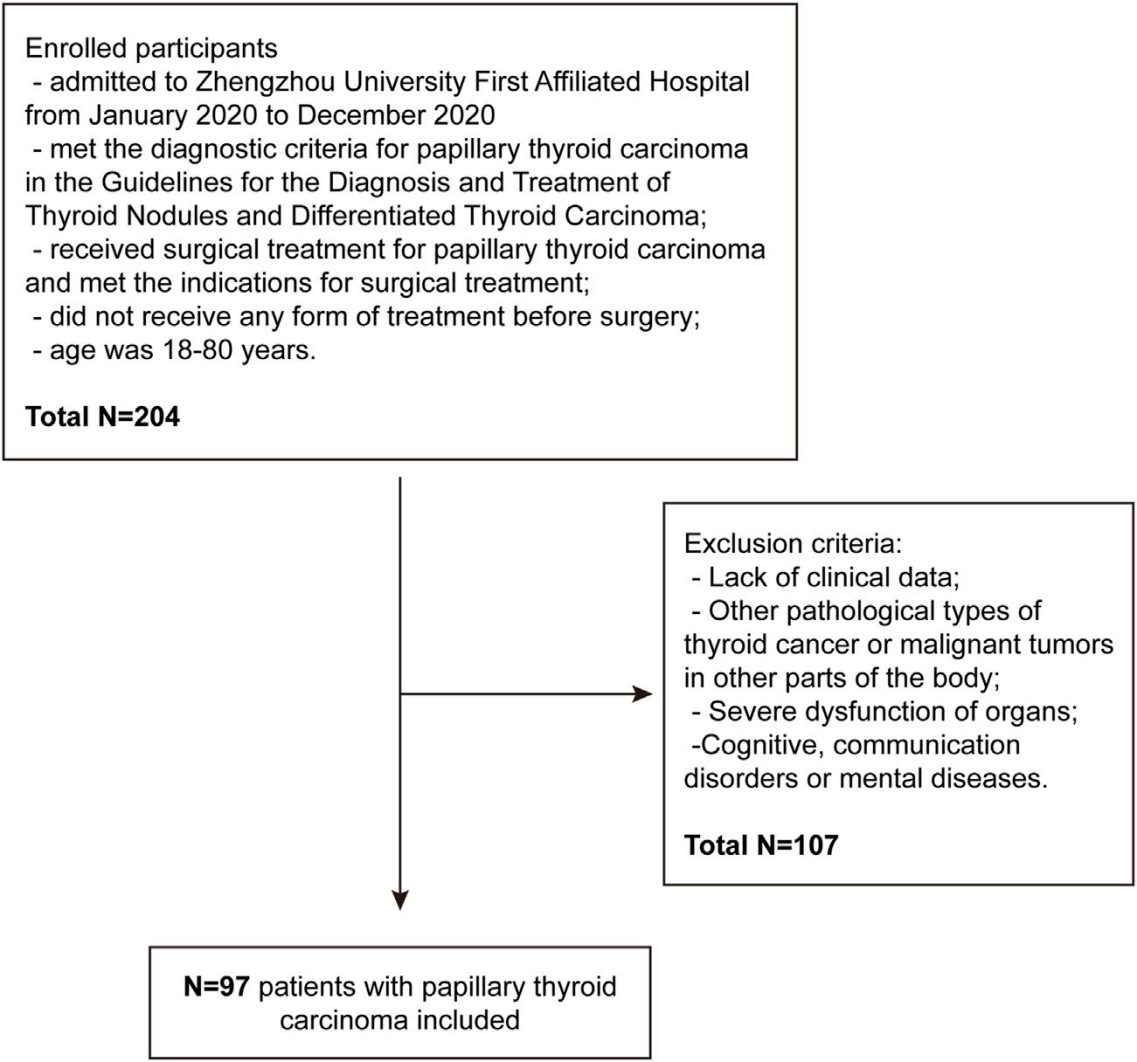
Study population flow diagram.

All human samples were obtained from the First Affiliated Hospital of Zhengzhou University with appropriate ethics committee approval (NO. 2024-KY-1912). This study included 97 (27 males and 70 females) patients with PTC admitted to Zhengzhou University First Affiliated Hospital between January 2020 and December 2020. The patients were aged <45 years in 29 cases and ≥45 years in 68 cases; the tumor diameter was >1 cm in 51 cases and ≤1 cm in 46 cases; the degree of differentiation was low in 25 cases and middle to high in 72 cases; the TNM staging was I–II in 60 cases and III-IV in 37 cases; lymph node metastasis occurred in 38 cases while no metastasis occurred in 59 cases. The inclusion criteria were as follows: (1) Patients who met the diagnostic criteria for PTC based on the 2015 American Thyroid Association Management Guidelines for Adult Patients with Thyroid Nodules and Differentiated Thyroid Cancer (Haugen et al., 2016); (2) patients who received surgical treatment for PTC and met the indications for surgical treatment; (3) patients who did not receive any form of treatment before surgery; (4) patients aged 18–80 years, and those who signed informed consent form. The exclusion criteria were as follows: (1) Patients without clinical data; (2) patients with other pathological types of thyroid cancer or malignant tumors in other parts of the body; (3) patients with severe organ dysfunction; (4) patients with cognitive, communication disorders, or mental diseases. This study has been approved by the Ethics Committee of Zhengzhou University First Affiliated Hospital (Figure 1).

### 2.2 Clinical specimens

All specimens used in this study were obtained from a tumor tissue bank at the First Affiliated Hospital of Zhengzhou University. During the tissue bank sample collection procedure, samples were routinely divided into three parts for different treatments. Samples designated for freezing were snap-frozen in liquid nitrogen and stored in −80°C freezers. The specimens for fixation were washed in 10% formalin and embedded in paraffin wax. Embedded samples were stored at room temperature.

### 2.3 Tissue microarray (TMA)

The tissue TMA was created from paraffin-embedded blocks of 58 paired carcinoma and 58 adjacent cases. Donor blocks were prepared after a comprehensive evaluation of hematoxylin and eosin-stained slides. For each cancer case, one representative section of cancerous tissue and one of the adjacent non-cancerous tissues were selected after identifying representative tumor regions in each whole-mount slide. A single core (0.6 mm in diameter) was extracted from the selected section of each donor block, using a specific orientation, and subsequently placed into a pre-molded recipient paraffin wax block. Consecutive 4 mm-thick sections were excised from the recipient blocks and affixed onto adhesive-coated slides for immunohistochemistry (IHC) analysis.

What needs notice is that, out of the 97 collected samples, 58 cases were chosen for TMA and subsequent IHC analysis. The remaining 39 samples were excluded due to inadequate tissue quantity or poor histological quality, which could potentially affect the reliability of IHC staining and interpretation. We ensured that only well-preserved and representative tissue cores were included in the TMA to guarantee the accuracy and validity of the IHC results. Furthermore, we compared the age, gender distribution, and tumor stage between the 58 cases included in the TMA and the remaining 39 cases that were excluded. No significant differences were observed, indicating that the TMA cohort can adequately reflect the characteristics of the entire sample set. Thus, the selected 58 cases are representative of the overall sample population in terms of key demographic and clinical characteristics.

### 2.4 PGK1 IHC and image analysis

Tissue sections of PTC and adjacent normal tissues were deparaffinized using xylene, hydrated with graded ethanol, dehydrated with graded ethanol, and heat-fixed at 100°C for 20 min. Subsequently, they were washed, treated with 0.3% H_2_O_2_ to inactivate endogenous peroxidase activity, and blocked with 5% goat serum. A rabbit monoclonal antibody against human PGK1 (proteintech, United States, 17811-1-AP) was introduced and incubated at 4°C overnight. The following day, diaminobenzidine (DAB) was used for colorimetric detection, and hematoxylin and eosin were used for counterstains. The sections were dehydrated, dried, and mounted with neutral bone cement. Phosphate buffered saline (PBS) was used as a negative control, and the outcome was observed under an optical microscope.

Images were graded based on the proportion of stained tumor cells and the staining intensity. The proportion of stained cells was scored from 0 to 4+ by calculating the percentage of positive to total epithelial cells in an area covering 25% of the tumor. 0 for 0% positive, 1+ for ≤25%, 2+ for >25% but ≤50%, 3+ for >50% but ≤75%, and 4+ for >75%. The intensity of immunostaining was scored semi-quantitatively as 0 for no obvious yellow particles in epithelial cell plasma membrane or cytoplasm; one for weak (light yellow particles); two for moderate (moderately yellow particles); 3+ for strong (deep yellow particles). Two pathologists independently determined scores for all samples. When the score disparity exceeded two, the slides were re-examined until the pathologists reached a consensus. The percentages of positive cells and staining intensity from the same specimen were added. If the sum was ≥3, the staining of the specimen was taken as high group.

### 2.5 RNA isolation and quantitative real-time polymerase chain reaction (qRT-PCR)

The specimens were lysed using a Total RNA Isolation Reagent (TRIzol reagent) (Invitrogen). Total RNA was extracted following the manufacturer’s protocol. RNA was then analyzed for purity by measuring the absorbance ratio at 260 and 280 nm using the NanoPhotometer Spectrometer (Thermo Fisher). Following the manufacturer’s protocol, cDNA libraries were synthesized using One-Step gDNA Removal and cDNA Synthesis SuperMix (Takara, Kyoto, Japan). A reverse transcription-polymerase chain reaction (RT-PCR) assay was conducted using 2× TB Green qPCR Master Mix (Takara), and the threshold cycle (Ct) value was measured. Beta Tubblin was used as the housekeeping gene for normalization, respectively. The comparative gene expression was calculated with the 2^−ΔΔCT^ method. All primers were synthesized by Genewiz Biotech (Nanjing, China). The primers used are listed in Table 1.

**TABLE 1:** Primers sequences.

Genes	Primers sequences
*Beta Tubblin*-forward	TGG​ACT​CTG​TTC​GCT​CAG​GT
*Beta Tubblin*-reverse	TGC​CTC​CTT​CCG​TAC​CAC​AT
*PGK1*-forward	TGG​ACG​TTA​AAG​GGA​AGC​GG
*PGK1*-reverse	GCT​CAT​AAG​GAC​TAC​CGA​CTT​GG

### 2.6 Immunoblot analysis

Total proteins were extracted from the specimens using radioimmunoprecipitation assay buffer (CST, #9806) supplemented with protease and phosphatase inhibitors (Thermo Fisher, #78441). The protein concentrations of the samples were determined by a microplate bicinchoninic acid protein assay kit (Beyotime Biotechnology, Shanghai, China). Equal amounts of protein (30 μg) were subjected to a 12% sodium dodecyl sulfate-polyacrylamide gel electrophoresis and transferred onto a nitrocellulose membrane for Immunoblot analysis. Antibodies used for Immunoblot include the following: PGK1 (1:1,000, Proteintech, United States, 17811-1-AP), Beta Tubblin (1:5,000, Proteintech, United States, 10094-1-AP). Blots were visualized using an LI-COR Odyssey imager, and the ImageJ analysis software (Version 1.49; NIH, United States) was used to determine each band intensity. Band densities of the indicated proteins were normalized to those of the corresponding loading controls.

### 2.7 Follow-up

The patients were followed up with outpatient check-ups and telephones until December 2023 as part of their postoperative monitoring. They were followed up every 3 months during the first year after surgery and subsequently every 6 months. The follow-up included computed tomography, magnetic resonance imaging, and whole-body bone scan examination. The incidence of tumor recurrence during the follow-up period was statistically analyzed. If the imaging examination suggested the original primary cancer site or other sites with new lesions, and the pathological examination confirmed that the lesion was a papillary thyroid carcinoma, it could be determined as recurrence.

### 2.8 UALCAN

UALCAN is a comprehensive and interactive web resource that provides easy access to publicly available cancer OMICS data (The Cancer Genome Atlas (TCGA), MET500, and Clinical Proteomic Tumor Analysis Con-sortium (CPTAC) databases and allows users to identify biomarkers or perform *in silico* validation of potential genes of interest (http://ualcan.path.uab.edu/index.html). Here, the mRNA expression of PGK1 with the 10-year survival rate of patients with THCA was evaluated using TCGA databases.

### 2.9 Collection of clinical data

The clinical data collected included the gender and age of the patients, underlying diseases history (hypertension and diabetes), tumor diameter, differentiation degree, TNM staging, TI-RADS grading, lymph node metastasis, surgical method, and dissection.

### 2.10 Statistical analysis

Statistical Package for the Social Sciences software (version 25.0; IBM Corp., United States) was used for data analysis for categorical variables, with PGK1 positive expression rate expressed as count and percentage. The chi-square test was used to analyze the relationship between PGK1 expression and the pathological characteristics and postoperative recurrence of patients with PTC. Univariate and multivariate Cox regression analyses were used to analyze the influencing factors on the prognosis of patients with PTC. Kaplan-Meier analysis was used to analyze the survival rate of patients with PTC followed up for 3 years, and the Log-Rank test was used to compare the survival rates of patients with high and low PGK1 expressions. A *P* < 0.05 was considered statistically significant.

## 3 Results

### 3.1 PGK1 is highly expressed in cancerous tissues of PTC

**FIGURE 2:**
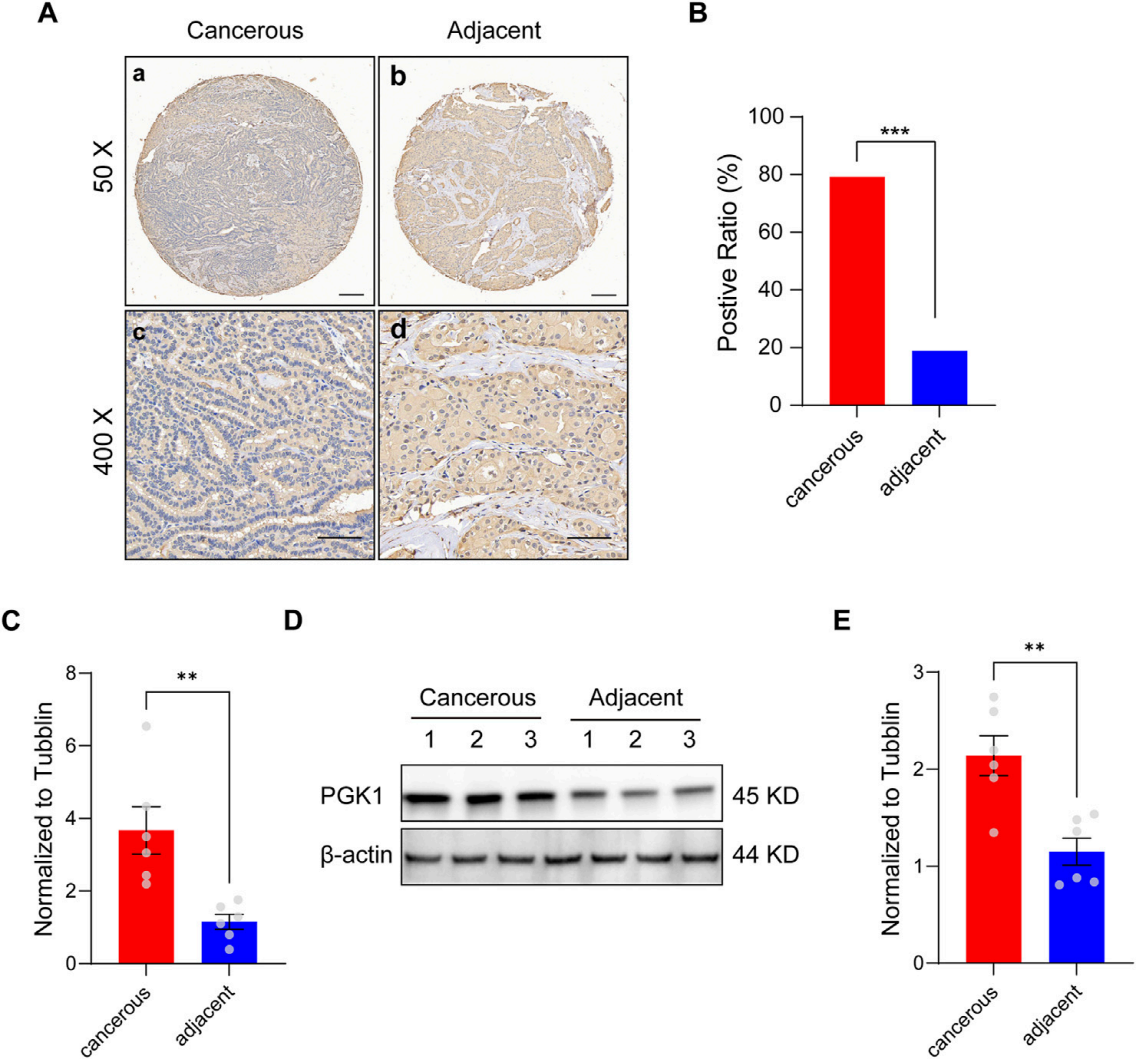
PGK1 is expressed strongly in cancerous tissue, but weakly in adjacent tissues. **(A)** lmmunohistochemical images of tissue microarray (TMA) analysis for PGK1 from a paired cancerous (a) and adjacent non-cancerous (b) case (magnifcation, ×50). Scale bar: 100 μm. Zoom image for PGK1 of a paired cancerous (c) and adjacent (d) case (magnifcation, ×400). Scale bar: 50 μm. **(B)** Positive ratio of PGK1 staining in TMA, 79.3% (46 of 58 cases) in cancerous samples and 18.9% (11 of 58 cases) in adjacent samples. **(C)** Quantitative RT-PCR analysis of the expression level of PGK1 in two groups. **(D)** Immunoblot of the protein level of PGK1 in two groups. **(E)** Immunoblot analysis of the protein level of PGK1 in two groups. Data are expressed as mean ± SEM. **P < 0.01, ***P < 0.001.

We performed TMA and IHC staining using paired samples from cancerous and adjacent non-cancerous tissues to investigate PGK1 expression in PTC. We analyzed 58 paired samples using TMA, and the number of samples that scored positive for PGK1 in cancerous (46/58, 79.3%) tissue was much higher than that for adjacent samples (11/58, 18.9%) (*P* < 0.05) (Figures 2A, B). Besides, we performed the Quantitative RT-PCR assay and immunoblot assay in two groups. The results demonstrated that the mRNA and protein levels of PGK1 were also significantly increased in cancerous tissues (Figures 2C–E). Meanwhile, we performed the IHC staining to investigated the PGK1 levels from 97 patients with PTC, and found that the PGK1 positive expression rate in cancerous tissues was much higher than that in adjacent tissues (*P* < 0.05; Table 2). All these results revealed that compared to the adjacent tissues, cancerous tissues displayed much higher signals for PGK1.

**TABLE 2:** Comparison of PGK1 positive expression in cancer tissues and adjacent tissues [n (%)].

Tissues	Number	Variable		χ^2^	*P*-values
		High	Low		
Cancerous tissues	97	72 (74.2%)	25 (25.8%)	55.901	**<0.001**
Adjacent tissues	97	20 (20.6%)	77 (79.4%)		

Significant *P*-values are in bold.

### 3.2 Relationship between PGK1 expression levels and clinicopathological characteristics of PTC

Patients with PTC were divided into PGK1 high group and low group based on the PGK1 expression levels and statistically analyzed according to each clinicopathological parameter. Between low and high PGK1 expression groups, age distribution, gender, history of hypertension, and diabetes exhibited no differences (*P* > 0.05). However, the percentage of high PGK1 expression levels in the cancer tissues of patients with low differentiation, TNM stages III–IV, lymph node metastasis, and tumor diameter ≥1.0 cm was greater than that of patients with high or moderate differentiation, TNM stages I–II, no lymph node metastasis, and tumor diameter <1.0 cm (*P* < 0.05; Table 3).

**TABLE 3:** Comparison of positive expression of PGK1 in PTC tissues with different clinicopathological features [n (%)].

Characteristics	Number	Variable		χ^2^	*P*-values
		High	Low		
Age (year)					
<45	29	24 (82.8%)	5 (17.2%)	1.574	0.210
≥45	68	48 (70.6%)	20 (29.4%)		
Gender					
Male	27	20 (74.1%)	7 (25.9%)	0.000	0.983
Female	70	52 (74.3%)	18 (25.7%)		
Hypertension					
Positive	30	20 (66.7%)	10 (33.3%)	1.298	0.255
Negative	67	52 (77.6%)	15 (22.4%)		
Diabetes					
Positive	15	11 (73.3%)	4 (26.7%)	0.007	0.931
Negative	82	61 (74.4%)	21 (25.6%)		
Metastasis					
Positive	38	35 (92.1%)	3 (7.9%)	8.958	**0.003**
Negative	59	37 (62.7%)	22 (37.2%)		
Differentiation					
Low	25	25 (100%)	0 (0%)	9.950	**0.002**
Medium–high	72	47 (65.3%)	25 (34.7%)		
TNM stage					
I ∼ II	60	36 (60.0%)	24 (40.0%)	14.750	**< 0.001**
III ∼ IV	37	36 (97.3%)	1 (2.7%)		
Tumor diameter (cm)					
<1.0	46	26 (56.5%)	20 (43.5%)	14.336	**< 0.001**
≥1.0	51	46 (90.2%)	5 (9.8%)		

Significant *P*-values are in bold.

### 3.3 Relationship between PGK1 expression levels and recurrence of PTC

We performed a 3-year follow-up to reveal the relationship between PGK1 expression levels and recurrence of PTC. The results indicated that 31 cases relapsed during 3 years of follow-up, with a recurrence rate of 31.96%. The PGK1 high expression rate in the cancer tissues of the recurrence group was higher than that of the cancer tissues of the non-recurrence group (*P* < 0.05; Table 4).

**TABLE 4:** Comparison of positive expression of PGK1 in cancer tissues between recurrence group and non-recurrence group [n (%)].

Recurrence	Number	Variable		χ^2^	*P-value*
		High	Low		
Positive	31	28 (90.3%)	3 (9.7%)	4.995	**0.025**
Negative	66	44 (66.7%)	22 (33.3%)		

Significant *P*-values are in bold.

### 3.4 Relationship between PGK1 expression levels and prognosis of PTC

**FIGURE 3:**
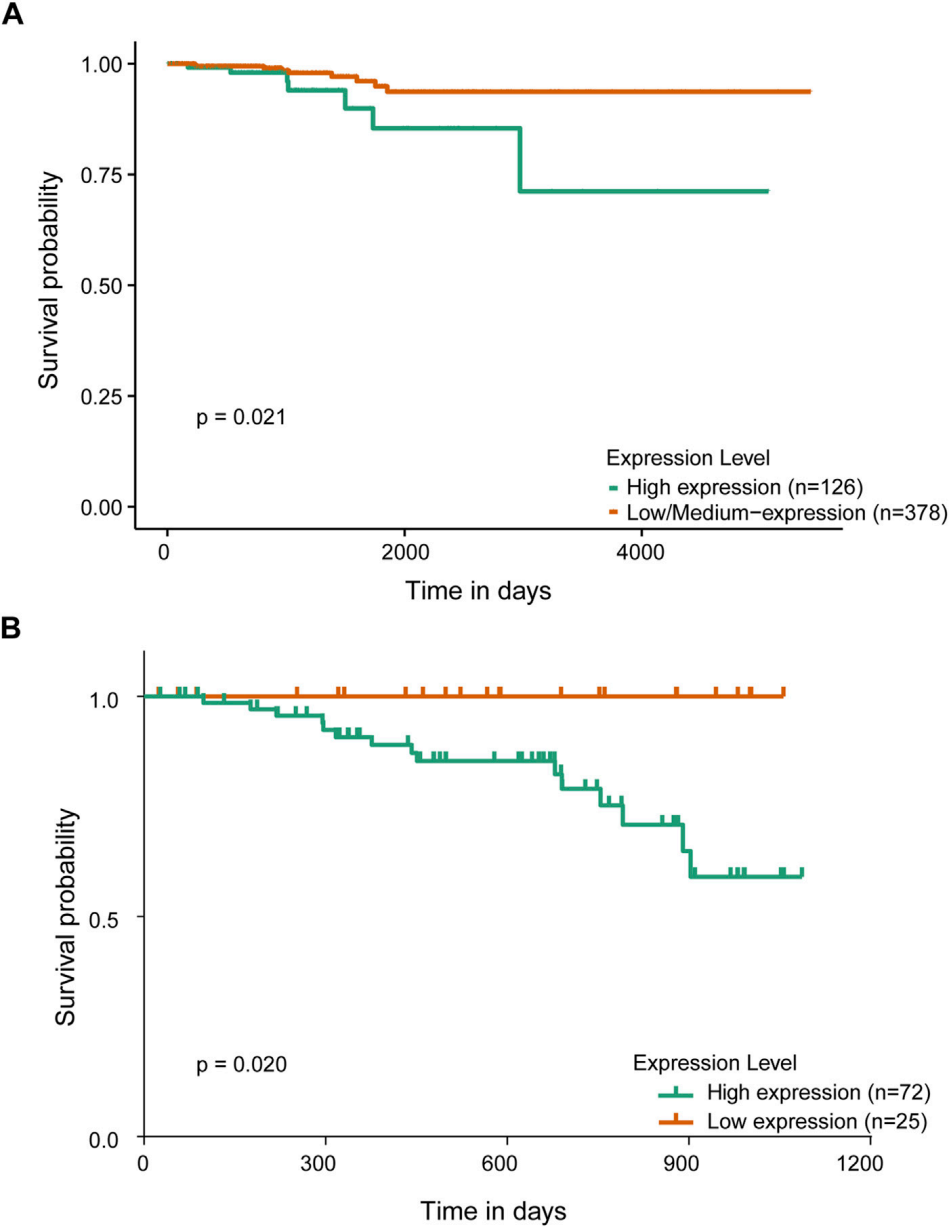
Relationship between PGK1 expression and prognosis in patients with papillary thyroid carcinoma. **(A)** Kaplan-Meier analysis of overall survival indicated the relationship between PGK1 expression and prognosis in patients with thyroid carcinoma in UALCAN database. **(B)** Kaplan-Meier analysis of overall survival indicated the relationship between PGK1 expression and prognosis in patients with PTC administrated in our datasets.

Subsequently, we investigated the correlation between PGK1 expression levels and the prognostic outcomes of patients with PTC. Initially, we analyzed the relationship between PGK1 expression levels and the 10-year survival rate of patients with thyroid carcinoma (THCA) in UALCAN database. Our findings revealed that patients with high PGK1 expression had a markedly diminished 10-year survival rate compared to those with low or medium PGK1 expression levels (Figure 3A). Within our dataset, of the 72 patients with elevated PGK1 expression levels, 57 survived, and 15 died, whereas all 25 patients with low PGK1 expression levels survived. The Kaplan–Meier survival curve indicated that the 3-year overall survival rate for the PGK1 high group was 79.20%, which was significantly lower than that of the PGK1 low group (100.00%) (*P* < 0.05; Figure 3B). These two datasets were consistent, collectively underscoring the association between elevated PGK1 expression and diminished overall survival rates. Concurrently, we compared the survival rate of the group with high PGK1 expression and the group with low PGK1 expression. As shown in Table 5, although there was no significant difference (*P* = 0.160), the survival rate of the population in the high-expression group of PGK1 was significantly lower than that in the low-expression group. However, we acknowledge that the relatively small sample size and the short-term follow-up period may limit the statistical power to detect potential differences in survival outcomes. In the future, we will collect more clinical samples and perform further analyses to assess the impact of potential selection bias.

**TABLE 5:** Univariate and multivariate Cox regression analysis of prognostic factors for PTC.

Characteristics	Number	Univariate analysis		Multivariate analysis	
		HR (95%CI)	P value	HR (95%CI)	P value
Age (year)	97				
<45	29	Reference		Reference	
≥45	68	0.255 (0.091–0.719)	**0.010**	0.262 (0.071–0.962)	**0.044**
Gender	97				
Male	27	Reference			
Female	70	1.674 (0.594–4.722)	0.330		
Hypertension	97				
Positive	30	Reference			
Negative	67	0.335 (0.075–1.487)	0.150		
Diabetes	97				
Positive	15	Reference			
Negative	82	0.034 (0.000–7.849)	0.223		
Metastasis	97				
Positive	38	Reference		Reference	
Negative	59	6.822 (1.920–24.241)	**0.003**	23.839 (1.832–310.259)	**0.015**
Differentiation	97				
Low	25	Reference		Reference	
Medium–high	72	17.041 (4.756–61.066)	**< 0.001**	93.545 (7.750–1129.165)	**< 0.001**
TMN stage	97				
I ∼ II	60	Reference		Reference	
III ∼ IV	37	4.804 (1.529–15.099)	**0.007**	0.005 (0.000–0.170)	**0.003**
Tumor diameter (cm)	97				
<1.0	46	Reference		Reference	
≥1.0	51	13.133 (1.725–99.989)	**0.013**	9.656 (0.712–130.940	0.088
PGK1 expression	97				
Low	25	Reference			
High	72	32.699 (0.251–4262.117)	0.160		

Significant *P*-values are in bold.

### 3.5 Univariate and multivariate Cox regression analysis of prognostic factors in patients with papillary thyroid carcinoma

The survival status of patients with PTC was considered the dependent variable, and gender, age, history of hypertension and diabetes, metastasis, differentiation degree, TNM staging, tumor diameter, and PGK1 expression levels were considered single factors for univariate Cox regression analysis. The results demonstrated that age <45 years, metastasis, poor differentiation, TNM staging III–IV, and tumor diameter ≥1 cm were independent risk factors for poor prognosis of patients with PTC (*P* < 0.05). The survival status of patients with PTC was taken as the dependent variable, and the influencing factors with *P* < 0.05 in the univariate Cox regression analysis, including age, metastasis, differentiation degree, TMN staging, and tumor diameter, were subjected to multivariate Cox regression analysis. The results revealed that age <45 years, metastasis, poor differentiation, and TMN stages III–IV were independent risk factors for poor prognosis of patients with PTC (*P* < 0.05; Table 5).

## 4 Discussion

PTC is the most common subtype of malignant thyroid tumors, accounting for approximately 80%–90% of all thyroid cancers (Dong et al., 2015). PTC generally manifests as a slow-growing tumor with characteristic histological features, which include papillary structures and nuclear variations, including nuclear grooves and inclusion bodies (Liu et al., 2023). The disease predominantly affects middle-aged and young women and has a good prognosis, with 5-year survival rates generally exceeding 90%. The pathogenesis of PTC is complex and involves various genetic and environmental factors, including RET/PTC rearrangements, BRAF mutations, and non-coding RNA (Nikiforov and Nikiforova, 2011; Lin et al., 2022). Despite the generally favorable prognosis of PTC, some patients may experience recurrence and metastasis; therefore, accurate diagnosis, staging, and individualized treatment strategies are essential for improving patient survival and quality of life.

PGK1 is an essential enzyme in glycolysis, responsible for catalyzing the conversion of 1,3-diphosphoglycerate (1,3-BPG) into 3-PGA while producing one ATP molecule (Qian et al., 2019; Duncan et al., 2022). PGK1 is widely expressed across cell types, and its activity is essential for maintaining cell energy metabolism (Qiu et al., 2024). Recently, the role of PGK1 in tumor biology has received increasing attention (Chen et al., 2022; Zhang et al., 2023). Guo et al. reported that PGK1 expression level was closely related to endometrial cancer occurrence and development, and PGK1 high expression patients exhibited more severe disease progression and worse prognosis (Guo et al., 2018). Additionally, PGK1 protein expression level was significantly upregulated in breast cancer tissues and cells. Hao Nie et al. reported that O-acetylglucosamine can modify PGK1, and it can regulate cell glycolysis and the tricarboxylic acid (TCA) cycle to promote colon cancer tumor cell growth (Nie et al., 2020). In patients with glioma, PGK1 expression level is expected to be one of the indicators for evaluating their radiation sensitivity and prognosis (Sun et al., 2017). In patients with prostate cancer, the change in serum PGK1 level is closely related to the postoperative efficacy and survival of patients and has diagnostic value (Chen et al., 2018; Pérez-Gómez et al., 2023). In fact, PGK1 exhibits remarkable tissue specificity across malignancies, especially in PTC. Studies shown that PTCSC3 inhibits aerobic glycolysis and proliferation of PTC by directly interacting with PGK1, which may be a potential therapeutic target for PTC treatment (Jiang et al., 2021). Besides, Xiang et al. found that the expression level of PGK1 is inversely correlated with the expression of FAM111B in clinical TC patient specimens, which promotes the growth, migration, invasion and glycolysis of PTC (Zhu et al., 2022). Consequently, PGK1 is considered an important molecular marker of tumor occurrence and progression, and its role in tumor metabolic regulation provides a new perspective for cancer diagnosis and treatment.

Several potential mechanisms may contribute the role of PGK1 in tumor progression. One plausible mechanism is that PGK1 may influence tumor biological behavior by regulating metabolic pathways. As a key enzyme in glycolysis, PGK1 plays a central role in the Warburg effect, which is a hallmark of cancer metabolism (Fukushi et al., 2022). By modulating glycolytic flux and the production of metabolic intermediates, PGK1 could potentially affect tumor cell proliferation, survival, and invasion (He et al., 2019). Another potential mechanism is that PGK1 may exert its effects through interactions with signaling pathways. PGK1 has been shown to interact with various signaling molecules and may participate in pathways such as the PI3K/AKT/mTOR pathway, which is frequently dysregulated in PTC (Li et al., 2025). The PI3K/AKT/mTOR pathway is a critical regulator of cell growth, proliferation, and survival, and its activation has been linked to poor prognosis in PTC patients (Bo et al., 2024). We speculate that PGK1 may enhance the activation of this pathway, thereby promoting tumor progression. Furthermore, PGK1 may also contribute to tumor progression by influencing the tumor microenvironment, For example, PGK1 could affect the production of extracellular matrix components or modulate the activity of matrix metalloproteinases, which are involved in tumor invasion and metastasis (Du et al., 2022). Additionally, PGK1 may interact with immune cells within the tumor microenvironment to modulate immune responses and create a more favorable environment for tumor growth (Zhang et al., 2018). Future studies is needed to validate these potential mechanisms.

This study aimed to investigate the role of PGK1 expression in PTC and its effects on prognosis. We derived a series of significant conclusions by comparing PGK1 expression differences between cancerous tissues and adjacent tissues, examining the correlation between PGK1 expression and clinicopathological features, and combining univariate and multivariate Cox regression analysis. This study demonstrated that the PGK1 positive expression rate in PTC cancer tissues is higher than that in adjacent tissues, which is consistent with the key role of PGK1 in the tumor glycolytic process. Additionally, high PGK1 expression is significantly associated with poor prognostic features, including low differentiation, advanced TNM staging, lymph node metastasis, and tumor diameter ≥1.0 cm, reinforcing the critical role of PGK1 in the progression of thyroid papillary carcinoma. The analysis revealed that the positive expression rate of PGK1 in the cancer tissues of the recurrence group was significantly higher than that in the non-recurrence group, indicating that PGK1 expression level may serve as a potential biomarker for forecasting the recurrence of thyroid papillary carcinoma after surgery. The Kaplan–Meier survival curve analysis further confirmed that the positive PGK1 expression was significantly negatively correlated with the 3-year overall survival rate of patients with PTC, providing strong evidence for PGK1 as a prognostic marker. The univariate Cox regression analysis revealed that age <45 years, lymph node metastasis, low differentiation, advanced TNM staging, and tumor diameter >1 cm were independent risk factors for the poor prognosis of patients with PTC. These factors are closely associated with the biological behavior and therapeutic response of PTC and provide an essential reference for clinical treatment decision-making.

Based on our results, we believed that PGK1 can work as a potential complementary marker to the genetic markers like BRAF and TERT in predicting PTC progression. As a hallmark of cancer metabolism, the overexpression of PGK1 in PTC may not only reflect metabolic reprogramming but also influence tumor progression through various downstream signaling pathways. In contrast, BRAF V600E and TERT promoter mutations are genetic alterations that are strongly associated with PTC development and prognosis (Yu et al., 2023). While these genetic markers provide valuable information about the tumor’s genetic landscape, PGK1 expression levels may offer additional insights into the tumor’s metabolic state and functional behavior. Integrating multiple biomarkers can enhance the predictive power and provide a more holistic assessment of tumor biology (Gibney et al., 2016). By combining PGK1 expression levels with BRAF V600E and TERT promoter mutation status, we may achieve a more accurate prediction of PTC patient outcomes and better stratify patients for personalized treatment strategies.

This study has some limitations. First, the sample size was relatively small, which may affect the generalizability and external validity of the results. Second, this study did not examine the relationship between PGK1 expression and treatment response in patients with PTC. Future studies should investigate the impact of PGK1 expression levels on treatment strategies for patients with PTC. Additionally, this study neglected other possible molecular markers that may affect prognosis, and future studies should use multi-omics approaches to comprehensively evaluate prognosis in patients with PTC.

## 5 Conclusion

In conclusion, this study demonstrated the expression characteristics of PGK1 in PTC and its correlation with clinical and pathological features and prognosis. High expression of PGK1 is associated with poor prognostic features of PTC and may be a potential biomarker for predicting postoperative recurrence of PTC. These findings provide a new perspective on prognostic evaluation and treatment strategy selection for PTC and direction for future research. Future studies should further confirm the clinical significance of PGK1 as a prognostic marker and investigate its potential application in treating PTC.

## Data Availability

The raw data supporting the conclusion of this article will be made available by the authors, without undue reservation.

## Abbreviations

PTC, Papillary Thyroid Carcinoma; PGK1, Phosphoglycerate kinase 1; 1,3-BPG, 1,3-diphosphoglycerate; 3-PGA, 3-phosphoglycerate; O-GlcNAc, O-acetylglucosamine; TCA, Tricarboxylic Acid; DAB, Diaminobenzidine; PBS, Phosphate buffered saline; IHC, Immunohistochemistry.
